# Dopamine adjusts the circadian gene expression of *Per2* and *Per3* in human dermal fibroblasts from ADHD patients

**DOI:** 10.1007/s00702-021-02374-4

**Published:** 2021-07-18

**Authors:** Frank Faltraco, Denise Palm, Adriana Uzoni, Lena Borchert, Frederick Simon, Oliver Tucha, Johannes Thome

**Affiliations:** Department of Psychiatry and Psychotherapy, University Medical Centre Rostock, Gehlsheimer Str. 20, 18147 Rostock, Germany

**Keywords:** ADHD Dopamine, Human dermal fibroblasts, Circadian rhythm

## Abstract

A link between dopamine levels, circadian gene expression, and attention deficit hyperactivity disorder (ADHD) has already been demonstrated. The aim of this study was to investigate the extent of these relationships by measuring circadian gene expression in primary human-derived dermal fibroblast cultures (HDF) after dopamine exposure. We analyzed circadian preference, behavioral circadian and sleep parameters as well as the circadian gene expression in a cohort of healthy controls and participants with ADHD. Circadian preference was evaluated with German Morningness-Eveningness-Questionnaire (D-MEQ) and rhythms of sleep/wake behavior were assessed via actigraphy. After *ex vivo* exposure to different dopamine concentrations in human dermal fibroblast (HDF) cultures, the rhythmicity of circadian gene expression (*Clock, Bmal1, Per1-3, Cry1*) was analyzed via qRT-PCR. We found no statistical significant effect in the actigraphy of both groups (healthy controls, ADHD group) for mid-sleep on weekend days, mid-sleep on weekdays, social jetlag, wake after sleep onset, and total number of wake bouts. D-MEQ scores indicated that healthy controls had no evening preference, whereas subjects with ADHD displayed both definitive and moderate evening preferences. Dopamine has no effect on *Per3* expression in healthy controls, but produces a significant difference in the ADHD group at ZT24 and ZT28. In the ADHD group, incubation with dopamine, either 1 µM or 10 µM, resulted in an adjustment of *Per3* expression to control levels. A similar effect also was found in the expression of *Per2*. Statistical significant differences in the expression of *Per2* (ZT4) in the control group compared to the ADHD group were found, following incubation with dopamine. The present study illustrates that dopamine impacts on circadian function. The results lead to the suggestion that dopamine may improve the sleep quality as well as ADHD symptoms by adjustment of the circadian gene expression, especially for *Per2* and *Per3*.

## Introduction

Dopamine is produced by dopaminergic neurons in the brain from aminoacid tyrosine, which is converted into dihydroxyphenylalanine (DOPA) by a rate-limiting enzyme, tyrosine hydroxylase (TH) (Tekin et al. 2014). Dopamine β hydroxylase (DBH) is an enzyme responsible for the conversion of dopamine into catecholamine neurotransmitter noradrenaline (Catelas et al. 2020). Circadian variations in the activity of tyrosine hydroxylase and dopamine β hydroxylase were observed in the rat brain stem and *Per1b* mutant zebrafish (Cahill and Ehret 1981; Huang 2015).

After biosynthesis, dopamine is packaged and stored into a synaptic vesicle by the vesicular monoamine transporter 2 (VMAT2) (Roeder 2002). A plasma membrane protein dopamine transporter (DAT), encoded by *SLC6A3* gene, controls both extracellular and intracellular concentrations of dopamine (McHugh and Buckley 2015; Salatino-Oliveira et al. 2018). Dopamine neurotransmitters bind to five subtypes of dopamine receptors: D_1_, D_2_, D_3_, D_4_, and D_5_, divided into two major subclasses: D-1-like and D-2-like members of the G-protein coupled receptor family (Beaulieu et al. 2015; Xin et al. 2019). When administered in critical care and emergency settings, dopamine acts as a non-selective drug and an agonist of α-, and β-adrenergic receptors (Farzam et al. 2020). Infused at low doses, between 0.5 and 3.0 μg/kg/min, dopamine increases diuresis, splanchnic blood flow and natriuresis. At higher doses than 3 μg/kg/min dopamine stimulates β-adrenergic receptors increasing cardiac inotropy and chronotropy, whereas doses higher than 7 μg/kg/min result in α-adrenergic stimulation with peripheral and splanchnic vasoconstriction (MacGregor et al. 2000). In critically ill new-born infants, plasma dopamine concentrations range from 0.5 ng/ml to almost 70 ng/ml, at an infusion rate of 4–8 μg/kg/min (Padbury et al. 1990). In hemodynamically stable children, the half-lives of distribution and elimination were 1.8 min and 26 min, respectively. The apparent volume of distribution was 2952 ± 2332 mL/kg, and clearance rate was 454 ± 900 mL/kg/min (Eldadah et al. 1991). In healthy male volunteers, injected with 3 μg/kg/min infusions for 90 min, steady-state dopamine concentrations varied from 1880 to 18,300 ng/l. After 10 min of dopamine infusion at 10 μg/kg/min plasma concentrations of dopamine varied from 12,300 to 201,500 ng/l, suggesting intra-individual and inter-individual variability in dopamine distribution and metabolism (MacGregor et al. 2000).

Dopamine is degraded by monoamine oxidase (MAO) in the cytosol and catechol-O methyl transferase (COMT) in the surrounding glial cells (Meiser et al. 2013). Main degradation products of dopamine are reactive 3, 4-dihydroxyphenylacetaldehyd and homovanilic acid (HVA) (Eisenhofer et al. 2004). Dopamine and its metabolites are metabolized by phase II conjugation reactions (Uutela et al. 2009).

Dopamine plays important roles in executive function, motor control, motivation, arousal, reinforcement, and reward through signaling cascades (Awata et al. 2015; Blenau and Baumann 2001; Waddell 2013). Studies have suggested that dopamine mediates learned associations between stimuli and reward (Beeler and Kisbye Dreyer 2019; Steinberg et al. 2013). Dopamine is a key regulatory component of executive function in the prefrontal cortex, and dopaminergic dysfunction can result in impaired working memory (Klaus and Pennington 2019). In addition, dopamine dysfunction has been linked with the development of many psychiatric disorders. The symptoms and signs of schizophrenia have been linked to high levels of dopamine in specific areas of the brain and maintenance on antipsychotic drugs prevents relapse to a much greater extent than placebo up to 2 years of follow-up (Ceraso 2020; Seeman 2013). Recent studies show that dopaminergic receptor signaling is disrupted in dyskinetic Parkinsonian rats (Jones-Tabah, 2020) and dopamine is associated with prioritization of reward-associated memories in Parkinson's disease (Sharp et al. 2020). Restless legs syndrome and attention deficit hyperactivity disorder (ADHD) are also associated with decreased dopamine activity (Guo 2017; Volkow 2009).

ADHD is one of the most prevalent psychiatric disorders in children and adults, characterized by symptoms of inattention, hyperactivity, impulsivity, or combined type that produce impairment across cognitive, behavioral, and interpersonal domains (Kooij 2019). The results for some dopamine genes, such as dopamine receptor D_4_ and D_5_, dopamine transporter (DAT) and DBH confirm the heredity of ADHD syndromes (Paclt et al. 2005). Patients with ADHD showed a reduction in dopamine synaptic markers associated with symptoms of inattention as shown in the dopamine reward pathway of participants with ADHD (Volkow et al. 2009). Medin et al. (2013) report low dopamine D_5_ receptor density in hippocampus in an animal model of ADHD (Medin et al. 2013). Dopamine genes, its signaling and metabolism are linked with the pathophysiology of ADHD (Barkley et al. 2019).

ADHD patients often display circadian abnormalities including sleep problems (Coogan et al. 2016; Fisher et al. 2014; Gau et al. 2007). ADHD has been linked with disturbances in chronotype, particularly increased eveningness in children aged 7–12 years (Durmus et al. 2017). Patients with diagnosed ADHD show shorter average sleep durations (Boonstra et al. 2007) and frequent nocturnal awakenings (Sobanski et al. 2008).

Dopamine is involved in the regulation of sleep as regulator of the sleep/wake cycle, exerting a potent wake-promoting activity (Eban-Rothschild et al. 2018). In addition, dopamine is linked to circadian rhythmicity and extreme light sensitivity of circadian entrainment (Hirsh et al. 2010). Circadian molecular clock machinery mechanism consists of an interconnected series of transcriptional-translational feedback loops involving Circadian Locomotor Output Cycles Kaput (CLOCK) and Brain and Muscle Arnt-like Protein 1 (BMAL1) that heterodimerize and promote transcription of the *Period* (*Per1/2/3*) and *Cryptochrome* (*Cry1/2*) genes (Buhr and Takahashi 2013). Circadian rhythms are driven by the central pacemaker in the mammalian brain, the suprachiasmatic nucleus (SCN) of the hypothalamus, also entrained in peripheral cells including fibroblasts cultured *in vitro* (Balsalobre 1998; Schibler and Sassone-Corsi 2002). Our research group observed circadian gene alterations at the molecular level of human dermal fibroblasts derived from human individuals (ADHD and control subjects) (Coogan 2019). Human-derived fibroblasts provide an advantageous model to study circadian rhythmicity as well as the influence of drugs on circadian gene expression (Faltraco et al. 2020). Circadian clocks are linked with the regulation of neurotransmitter systems. Disruptions to these circadian clocks can effect cognitive functions in various diseases with altered neurotransmitter signalling (Kiehn et al. 2019).

Many essential psychiatric medications modulate the effects of dopamine, such as levodopa (L-DOPA) for Parkinson's Disease, and dopamine antagonists like antipsychotic and anti-nausea agents.

The psychostimulant methylphenidate and the selective norepinephrine transporter inhibitor atomoxetine, are two of the most frequently prescribed medications for ADHD. Methyphenidate blocks DAT, increasing levels of extracellular dopamine in the prefrontal cortex and striatum, as well as increasing concentrations of norepinephrine in the prefrontal cortex and hippocampus (Kuczenski and Segal 2002; Volkow 2001, 2002).

Atomoxetine increases extracellular levels of norepinephrine and dopamine in prefrontal cortex of rats, without effecting dopamine concentrations in striatum or nucleus accumbens (Bymaster 2002). Ide et al. observed that low-dose methylphenidate alters the reward system in wild-type mice via dopamine transporter inhibition. Dopamine transporter knockout mice do not exhibit such alterations. High-dose methylphenidate suppresses intracranial self-stimulation, suggesting the possibility that methylphenidate treatment does not increase the risk of drug dependence (Ide et al. 2018). Using HDF as a model, Coogan et al. (2019) report alterations in the expression of *Per2* and *Cry1* between subjects with ADHD without medication, compared to ADHD subjects taking medication, or controls. *Clock* gene expression was also altered in pharmaceutically treated ADHD subjects. Analysis of fibroblasts transfected with a *Bmal1:luc* reporter demonstrated changes in the timing of the peak expression across the three groups. Behavioral data that indicate that patients with ADHD using ADHD medication have lower relative amplitudes of diurnal activity rhythms, lower sleep efficiency and more nocturnal activity (Coogan et al. 2019).

Based on the assumption of the effectiveness of dopamine for the treatment of ADHD, its influence on the expression of the core circadian genes (*Clock, Bmal1, Per1-3, Cry1-2*) is hypothesized. Therefore, in this study, the influence of dopamine on circadian rhythmicity are investigated in vitro.

## Materials and methods

### Participant selection criteria

Ethical approval for the conduct of the study, including obtaining human dermal biopsy samples, was given by the ethical review committee of Rostock University (Registration-number: A2013-159) and written consent was obtained from each study participant. The study was conducted according to the ethical guidelines of the Declaration of Helsinki.

ADHD patients and healthy controls participating in the study were recruited via the Department of Psychiatry and Psychotherapy, University Medical Centre Rostock. All ADHD patients were diagnosed by experienced psychiatrists in advance. The healthy control group was recruited of acquaintances of people involved in the study.

Human dermal fibroblasts (HDF) were obtained from skin biopsies from dorsal forearm from ADHD patients and healthy control volunteers. Only adult individuals, able to give informed consent, were included. Healthy controls without a history of childhood and adult ADHD were matched for sex and age. Patients with more severe than ADHD symptoms, were excluded, as were shift workers. Screening for ADHD symptoms was done by using the WURS-k (Wender Utah Rating Scale) as well as assessment of symptoms according to DSM-IV and ICD-10 criteria. Additional, the following psychometric tests were used to confirm ADHD diagnosis: SKIDI and II (Structured clinical interview), DIVA 2.0 (Structured diagnostic interview), CAARS (Conners' Adult ADHD Rating Scales) and PSQI (Pittsburgh Sleep Quality Index). The IQ of the healthy control group and volunteers with ADHD diagnosis were measured using MWT (Multiple-Choice Word Test). The chronotype of the participants were determined by the D-MEQ (Morning-Eveningness-Questionnaire, German Version). No special cognitive testing was implemented in the study.

Comorbidities were observed: 33.3% of participants with ADHD diagnosis has additionally adipositas, 8.3% has additionally addiction disorder, and 25.1% has additionally affective disorder. The remaining participants with ADHD diagnosis has no comorbidities.

The four manuscripts of this special issue dealing with circadian rhythmicity describe unique research questions (Faltraco et al. 2021a, b; Palm et al. 2021). Although some samples have been used for more than one research question, the overall sample composition differs from each other and thus is different for each study. Experiments differ substantially in their conditions, thus, they each investigate unique cellular biochemical pathways.

### Actigraphy

To obtain objective measures of participants’ sleep and circadian rhythm function, the rest–activity pattern of participants was recorded using wrist-worn actigraphs (Actiwatch 2, Philips Respironics, USA). Actigraphs were worn on the non-dominant wrist for a period of at least 7 consecutive days. The recording interval of the device was set at 60-s epochs. Data occurring before the first and after the final midnight of each record were excluded, ensuring at least 6 complete days for each participant, with a complete weekend included in each record.

### Tissue isolation and fibroblast cell culture

Human dermal fibroblasts (HDF) were isolated and cultured as described previously (Takashima 1998). Fibroblasts were cultivated (37 °C, 5% CO_2_) in Dulbecco's Modified Eagle Medium DMEM (Gibco, Thermo Fisher, UK) /1 mg/ml Liberase TM (Roche, Germany) containing 100 units/ml penicillin, 100 µg/ml streptomycin (Gibco, Thermo Fisher, UK) and 10% fetal bovine serum FBS (Gibco, Thermo Fisher, UK).

### Measurement of cell viability

Upon confluency of the respective primary fibroblast cell culture from each participant, cells were incubated with 0 µM, 1 µM and 10 µM dopamine (Vitamaze, Germany). Following 24 h cell viability was measured using the Trypan Blue Exclusion Test (Strober 2015).

### Measurement of circadian gene expression

Upon confluency of the respective primary fibroblast cell culture from each participant, eight culture flask replicates were prepared and cells were incubated with either 1 µM or 10 µM dopamine (Vitamaze, Germany). Cultures without dopamine were used as a negative control. After 24-h of incubation, the cells were synchronized with 100 nM dexamethasone (Sigma-Aldrich, Germany) for 30 min. Samples were harvested every fourth hour after synchronization for a period of 28-h in solution D (4.5 M guanidinium thiocyanate, 0,5% sodium-N-lauryl sarcosine, 25 mM tri-sodium citrate, 0.1 M betamercaptoethanol) and stored at -70 °C. Total RNA was isolated and purified with RNeasy Plus Mini Kit (Qiagen, Germany) as well as subjected to reverse transcription using the Superscript III First-Strand Synthesis System (Invitrogen, Germany). Gene expression of *Clock, Bmal1, Per1, Per2, Per3* and *Cry1* as well as housekeeping genes (*Rpl13A, Rpl19A, GAPDH*) was measured by real-time quantitative reverse transcriptase polymerase chain reaction (qRT-PCR) with CFX Connect™ Real-Time PCR Detection System (Biorad, Germany). The oligonucleotide sequences are presented in Table 1. All primers were purchased from Eurofins (Alameda, CA). The qRT-PCR was performed in 96-well 0.1-ml thin-wall PCR plates (Applied Biosystems) in the CFX Connect™ Real-Time PCR Detection System (Biorad, München, Germany) as previously described (Coogan 2019).

**Table 1:** Oligonucleotides for qRT-PCR to measure circadian gene expression

Gene	Forward primer (5′–3′)	Reverse primer (5′–3′)
*Clock*	CCAGCAGTTTCATGAGATGC	GAGGTCATTTCATAGCTGAGC
*Bmal1*	AAGGATGGCTGTTCAGCACATGA	CAAAAATCCATCTGCTGCCCTG
*Per1*	TGGGGACAACAGAACAGAGAA	AGGACACTCCTGCGACCA
*Per2*	GTATCCATTCATGCTGGGCT	TCGTTTGAACTGCGGTGAC
*Per3*	TCAGTGTTTGGTGGAAGGAA	TCTGGGTCAGCAGCTCTACA
*Cry1*	CACGAATCACAAACAGACGG	TACATCCTGGACCCCTGGT
*RPL13a*	GCCAGAAATGTTGATGCCTT	AGATGGCGGAGGTGCAG
*RPL19a*	GTGGCAAGAAGAAGGTCTGG	GCCCATCTTTGATGAGCTTC
*GAPDH*	GAAGGTGAAGGTCGGAGT	GAAGATGGTGATGGGATTTC

### Statistical methods

Circadian gene expression data were tested for significant circadian rhythmicity, using CircWave v. 1.4 software (generated by Dr.Roelof Hut; www.euclock.org) to determine the best-fitting linear harmonic regression with an assumed period of 24-h and with α set at 0.05. The center-of-gravity of each best-fitting waveform in CircWave was used as the circadian acrophase, and the associated estimation error was used as the SD. Inferential statistics were carried out in SPSS (IBM Corporation).

Actigraphic data were analyzed via MANCOVAs, with age, sex and in some cases ADHD symptom severity included in the model as co-variates.

qRT-PCR clock gene data were analyzed via ANOVA. For all inferential tests, *P* < 0.05 was used to indicate a statistically significant groupwise difference. Sample sizes were calculated via GPower 3.1 software; for correlations, the assumptions used were significance level of *α* = 0.05 and the power of 0.8 for 2 groups (ADHD, HC) with 3 measures (0 µM, 1 µM and 10 µM dopamine). Although research in this field is generally scarce, we assumed that the influence of dopamine on the circadian gene expression will have an effect size *d*’ = 0.5, returning a required total sample size of 21. Taking into consideration an expected drop-out rate, *n* = 12 participants were allocated per each group. Data were analyzed via time series statistics adequately powered by 12 samples each, which in this statistical model is mathematically sufficient and thus representative (Menet et al. 2012; Thaben and Westermark 2016).

## Results

### Demographic data

Human dermal fibroblasts (HDF) were obtained via skin biopsy from volunteers with attention deficit hyperactivity disorder (ADHD) (8 men, 4 women; 45.08 ± 18.07 years, mean ± SD; BMI: 26.73 ± 4.48 kg/m^2^, mean ± SD), matched by healthy controls (HC) (4 men, 8 women; 41.50 ± 14.04 years, mean ± SD; BMI: 25.87 ± 5.42 kg/m^2^, mean ± SD). All participants completed the Multiple-Choice Word Test (IQ score: ADHD participants: 107.50 ± 10.91, mean ± SD, HC: 110.25 ± 9.32, mean ± SD), Morningness-Eveningness-Questionnaire, German Version (D-MEQ Score: ADHD participants: 44.33 ± 16.14, mean ± SD, HC: 58.83 ± 8.97, mean ± SD, *p* < 0.01) and Wender Utah Rating Scale, German Short Version (WURS-k Score: ADHD participants: 41.50 ± 14.94, mean ± SD; HC: 7.17 ± 8.19, mean ± SD, *p* < 0.0001). The demographic data are presented in Table 2.

**Table 2:** Demographic data

Demographic data	Healthy controls *n* = 12	ADHD *n* = 12
Age	41.50 ± 14.04 years	45.08 ± 18.07 years
Female	8 (66.7%)	4 (33.3%)
BMI	25.87 ± 5.42	26.73 ± 4.48
IQ-Score	110.25 ± 9.32	107.50 ± 10.91
D-MEQ	58.83 ± 8.97**	44.33 ± 16.14**
WURS-k-Score	7.17 ± 8.19***	41.50 ± 14.94***

*p < 0.01, ***p < 0.001

There were no significant differences in age, BMI, IQ or gender across the two study groups. D-MEQ scores indicated that ADHD patients displayed more definitive and moderate evening preference than HC. 58.3% of healthy participants displayed neutral preferences, whereas 25.0% had moderate morning preference, and 16.7% definite morning preference. 25.0% of ADHD participants displayed moderate morning preference, and 33.3% neutral preference. In the ADHD group the evening preference was represented by 25.0% definite evening and 16.7% moderate evening type. There were no participants with definitive morning preference in the ADHD group.

### Actigraphy

**Fig. 1:**
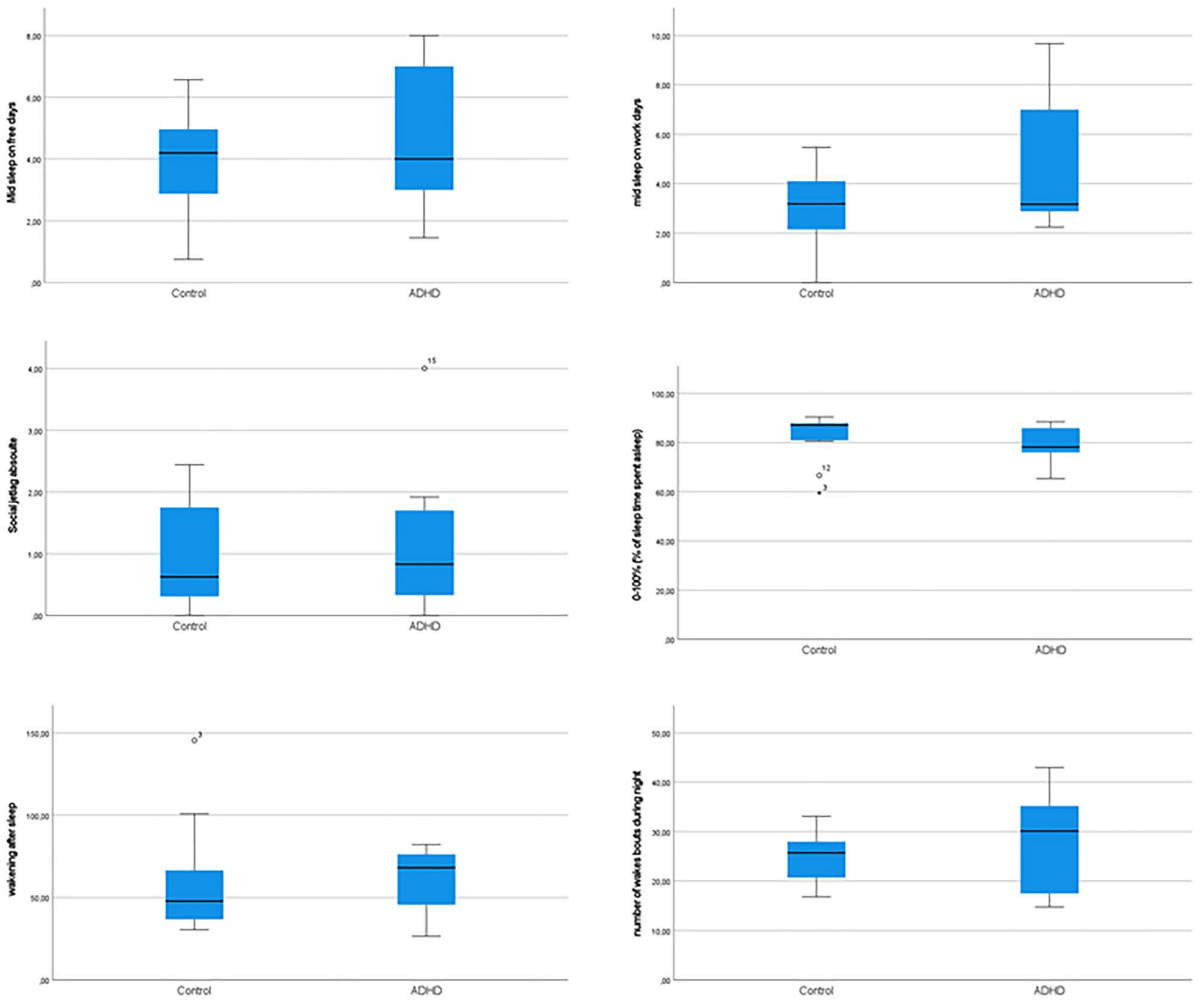
Actigraphic measures of mid-sleep of weekend days, mid-sleep of week days social jetlag, sleep efficiency, WASO (wakening after sleep onset) and total number of wake bouts are displayed as boxplots. Circles correspond to outer values and asterisks correspond to extreme values

Measures from the non-parametric circadian rhythm analysis were analyzed across the two groups, healthy controls and ADHD participants, in a MANCOVA with age and sex as co-variates. No statistically significant effect of group was observed (Pillai’s trace = 0.380; *F* = 1.226; *P* = 0.359; partial ETA squared = 0.380). No significant difference for mid-sleep on weekend days (*p* = 0.774), mid-sleep on weekdays (*p* = 0.169), social jetlag (*p* = 0.984), sleep efficiency (*p* = 0.833), WASO (wakening after sleep onset; *p* = 0.844) and total number of wake bouts (*p* = 0.425) was shown (Fig. 1). The measurements for three ADHD volunteers were not completed.

### Cell viability

The viability of the cultivated human dermal fibroblasts (HDF) after dopamine incubation was compared with HDFs without dopamine. The viability of cells treated with Dopamine (1 µM Dopamine: 91.067 ± 0.007, mean ± SD; 10 µM Dopamine: 90.204 ± 0.002, mean ± SD) was higher than compared to control cells without dopamine (0 µM Dopamine: 83.650 ± 0.009, mean ± SD). After incubation with dopamine the cell numbers increased by 147% for 1 µM Dopamine and 154% for 10 µM Dopamine, compared to the cells without dopamine.

### Circadian gene expression in human dermal fibroblasts

The expression profiles of circadian genes after incubation with 1.0 µM and 10.0 µM dopamine concentrations were examined in primary fibroblasts cultured from skin biopsies collected from ADHD and healthy participants and synchronized with dexamethasone. Cultures without dopamine were used as a negative control.

*Bmal1*, *Cry1* and *Per3* expression was strongly rhythmic in both groups (CircWave, *p* < 0.001). No rhythmicity was detected for *Clock* in both groups except for cultures incubated with 1 µM dopamine (CircWave, *p* < 0.05). In the ADHD group, 10 µM dopamine exposure dampened the rhythmicity of *Clock* (CircWave, *p* > 0.05). The same effect was observed for *Per1/2* genes in cultures from healthy controls (HC) (CircWave, *p* > 0.05). Dopamine shifted the *Clock* circadian acrophase to 20.00 ± 3.37 h (CircWave, mean ± SD) compared to 5.68 ± 3.37 h (CircWave, mean ± SD) in cultures without dopamine. In the ADHD group exposure to dopamine shifted the *Per1* circadian acrophase to 5.63 ± 2.22 h (CircWave, mean ± SD) compared to 7.07 ± 2.09 h (CircWave, mean ± SD) in cultures without dopamine.

**Fig. 2:**
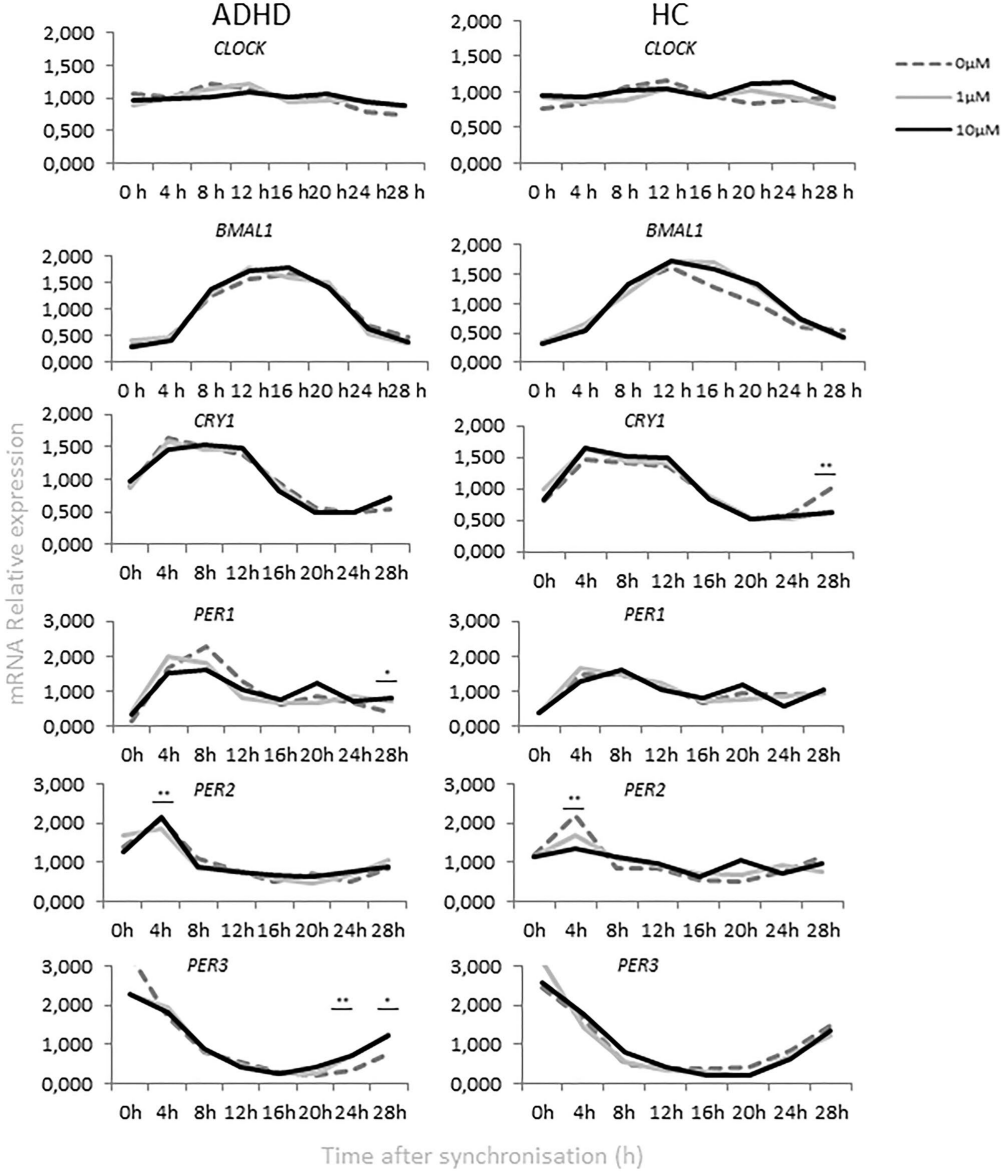
Relative mRNA gene expression of circadian genes in healthy controls and ADHD volunteers (0, 1.0, 10.0 µM Dopamine). *p < 0.05, **p < 0.01

**Fig. 3:**
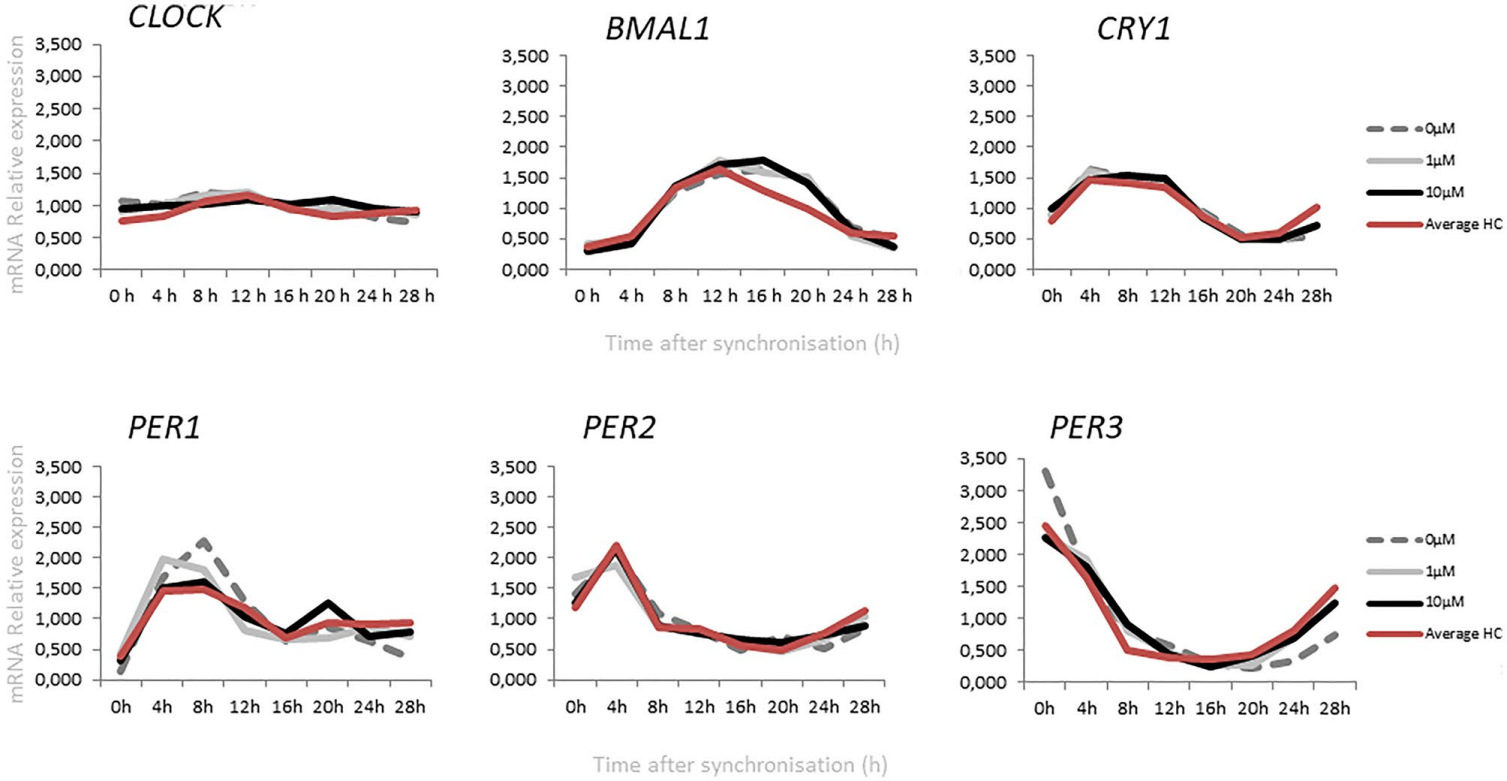
Relative mRNA gene expression of circadian genes in healthy controls (0 µm) and ADHD volunteers (0, 1.0, 10.0 µM Dopamine)

Differences of clock gene expression levels among study groups were assessed using one-way ANOVA (Fig. 2). When comparing the two study groups, ADHD and HC, one-way ANOVA revealed significant different *Per2* at ZT4 (*p* = 0.003, *F* = 4.075) and *Cry1* at ZT28 (*p* = 0.002, *F* = 4.451). A Bonferroni post hoc correction revealed a significant different expression of *Per2* (ZT4, *p* = 0.011) between the HC and ADHD cultures incubated with 10 µM dopamine. The expression and rhythm of *Per2* gene in the ADHD group resulted in an adjustment to the HC group after 10 µM dopamine incubation (Fig. 3). The expression of *Cry1* (ZT 28, *p* = 0.001) was different between HC and ADHD without dopamine incubation.

Gene expression in healthy participants revealed a statistical significant difference between cultures incubated with dopamine and negative controls (without dopamine incubation), as determined by one-way ANOVA for *Cry1* at ZT28 (*F* = 5.594, *p* = 0.008) and *Per2* at ZT4 (*p* = 0.043, *F* = 3.473). A Bonferroni post hoc correction revealed a significant lower *Cry1* expression in cultures incubated with 1 µM dopamine (*p* = 0.019) and 10 µM dopamine (*p* = 0.018) compared to HDF cultures without dopamine. 10 µM dopamine significant lowered the expression levels of *Per2* at ZT4 (*p* = 0.040) compared to the cultures without dopamine. One-way ANOVA revealed in the ADHD group statistical significant *Per2* expression at the same time-point, ZT4 (*p* = 0.024, *F* = 4.170) between samples incubated with 1 µM and 10 µM dopamine. In the ADHD group, statistical significant differences were observed at ZT24 and ZT28 between cultures incubated with dopamine and negative controls, particularly, for period genes *Per1* (ZT28, *F* = 5.103, *p* = 0.012) and *Per3* (ZT24, *F* = 7.703, *p* = 0.002; ZT28, *F* = 3.800, *p* = 0.033). A Bonferroni post hoc correction revealed a significant higher expression of *Per1* at ZT28 in cultures incubated with 1 µM dopamine (*p* = 0.049) and 10 µM dopamine (*p* = 0.016) compared to negative controls. The same effect was observed for *Per3* gene expression for the cultures incubated with 1 µM dopamine (ZT28, *p* = 0.039). 1 µM (*p* = 0.003) and 10 µM (*p* = 0.010) dopamine increased the expression of *Per3* at ZT24 compared to the negative controls.

## Discussion

In the present study, the incubation of human dermal fibroblast cultures with 1 µM dopamine induced the rhythmicity of *Clock* gene. This effect was not observed in cultures incubated with 0 and 10 µM dopamine. Preliminary results from our work has shown that *Clock* gene rhythmicity is also linked with the neurotransmitter norepinephrine. Norepinephrine is a known synchronizer of the circadian rhythm (Li and Cassone 2015; Maletic et al. 2017). This effect could suggest a potential link between the monoamines neurotransmitters and circadian rhythm pathways.

Hirsh et al. observed that dopamine plays an important role in the circadian behavior of *Drosophila melanogaster.* These drosophila mutants lacking tyrosine hydroxylase neural expression show weak circadian rhythmicity. The tyrosine hydroxylase rescue strain, deficient in neural dopamine also selectively shows a defect in circadian entrainment to low light levels (Hirsh et al. 2010). Under a light–dark cycle, tyrosine hydroxylase exhibited rhythmic patterns of transcription in chicken embryonic retinal cells (Lima et al. 2011). Low levels of dopamine also were observed in zebrafish mutants for the circadian gene *period1b* (Huang et al. 2015). These zebrafish mutants display hyperactivity, impulsivity-like and inattention-like behavior. Huang et al. found that the circadian clock regulates dopamine-related genes dopamine β hydroxylase and MAO. MAO is rhythmically expressed in wild-type larvae and upregulated in *per1b* mutant larvae (Huang et al. 2015). Kim et al. studied the influence of *Per2* in cases of methamphetamine addiction and observed that *Per2*-overexpressed mice presented lower dopamine levels compared to *Per2*-knockout mice, suggesting that *Per2* may influence the addictive effects of methamphetamine through the dopaminergic system (Kim, 2019). Bussi et al. studied interval timing (duration discrimination within the seconds-to-minutes range), which involves the dopaminergic–glutamatergic pathway. The authors suggested that the lack of dopamine rhythmicity under constant light is probably regulated by *Per2* and this could be responsible for impaired performance in the timing task in the mice model organism (Bussi et al. 2014). Hood et al. demonstrated a direct relationship between extracellular dopamine levels and the rhythm of expression of the clock protein PERIOD2 (PER2) in the dorsal striatum in male Wistar rat. The authors suggested that the rhythm of expression of PER2 depends on daily dopaminergic activation of D(2) dopamine receptors (Hood, 2010).

The study of Yokokura et al. suggests that microglial activation and dopamine D1 receptor reduction, as well as their aberrant interactions underpin the neurophysiological mechanism of ADHD (Yokokura, 2020). Volkow et al. demonstrated that a reduction in dopamine synaptic markers are associated with symptoms of inattention in the dopamine reward pathway of participants with ADHD. The D2/D3 receptor measures were correlated with attention implicating the dopamine reward pathway in the symptoms of inattention in ADHD (Volkow et al. 2009). Previous studies focused on genetics and environmental etiologies also proposed a relation between dopamine and ADHD (Braun et al. 2006; Swanson, 2007). Imaging studies observed that brain dopamine neurotransmission is disrupted in ADHD (Ernst et al. 1999; Lou et al. 2004; Rosa Neto et al. 2002; Volkow et al. 2007a, b).

To the best of our knowledge, until now, no studies have analyzed the influence of dopamine in human fibroblast cell cultures of healthy controls and volunteers with ADHD. The results of the present study illustrate that ADHD leads to alterations in the circadian rhythm. It demonstrates that dopamine impacts on circadian function, particularly the *Cry1, Per1/2/3* gene expression.

Dopamine showed no effect on *Per3* expression in healthy controls, but exhibited a significant difference in the ADHD group at ZT24 and ZT28 compared to samples without dopamine incubation. Incubation with dopamine, either 1 µM or 10 µM, result in an adjustment of *Per3* expression to the healthy controls (without dopamine incubation). Additionally, dopamine significantly reduces the *Per2* expression in cells of healthy controls. In the ADHD group, dopamine incubation results in an adjustment of *Per2* expression to healthy controls without dopamine incubation. These results lead to the suggestion that dopamine may improve the sleep quality as well as ADHD symptoms by adjustment of the circadian gene expression, especially for *Per2* and *Per3*.

It is to mention, that no special cognitive testing was implemented in this study. In addition, the participants of the ADHD group took no medication during the study. For further studies, a connection between circadian disturbances, cognitive deficits and the effect of medication would be suitable.

## Data Availability

Actiwatch 2, Philips Respironics, USA; CircWave v. 1.4 software (generated by Dr. Roelof Hut; www.euclock.org); SPSS (IBM Corporation).
